# Polymorphisms analysis for association between ADIPO signaling pathway and genetic susceptibility to T2DM in Chinese han population

**DOI:** 10.1080/21623945.2021.1978728

**Published:** 2021-10-12

**Authors:** Haibing Yu, Wei Hu, Chunwen Lin, Lin Xu, Hao Liu, Ling Luo, Rong Chen, Jialu Huang, Weiying Chen, Chen Yang, Danli Kong, Yuanlin Ding

**Affiliations:** a Department of Epidemiology and Medical Statistics, School of Public Health, Guangdong Medical University, Dongguan, Guangdong, China; b Key Laboratory of Prevention and Management of Chronic Kidney Disease of Zhanjiang City, Institute of Nephrology, Affiliated Hospital of Guangdong Medical University, Zhanjiang, Guangdong, China; c Shenzhen Center for Chronic Disease Control, Shenzhen, Guangdong, China

**Keywords:** Type 2 diabetes mellitus, adipo signalling pathway, single nucleotide polymorphisms

## Abstract

The aim of the present study is to explored the relationship between ADIPO signalling pathway and T2DM, to provide clues for further study of the pathogenesis of T2DM and to determine the possible drug targets. This study employed a case-control study design. Twenty-three single nucleotide polymorphisms (SNPs) of 13 genes in the selected ADIPO signalling pathway were genotyped by SNPscan^TM^ kit. All statistical analysis was performed by SPSS 25.0, PLINK 1.07, R 2.14.2, Haploview 4.2, SNPstats, and other statistical software packages. In the association analysis based on a single SNPs, rs1044471 had statistical significance in the overdominant model without adjusting covariates. Rs1042531 had statistical significance in the overdominant model. Rs12718444 had statistical significance in the recessive model. There was a linkage disequilibrium between the loci within 9 genes, and the two loci in RXRA gene did not form blocks. Four kernel functions were used for SNPs set analysis based on ADIPO signalling pathway showed that there was no statistical significance whether covariates were added or not, P>0.05.According to our research results, it is found that some single nucleotide polymorphisms (*ADIPOR2* rs1044471, *PCK1* rs1042531, *GLUT1* rs12718444) in the adiponectin signalling pathway may be associated with T2DM

## 1. Introduction

The 9th edition of the Diabetes Map released by the International Diabetes Federation shows that the prevalence of diabetes among adults aged 20–79 in the world reached 9.3% in 2019, indicating that about 463 million adults worldwide suffer from diabetes; China has the largest number of diabetes patients in the world, with an estimated 116.4 million, and is expected to reach 147.2 million by 2045 [1]. T2DM is a metabolic disease caused by the interaction of environmental factors and genetic factors [2]. T2DM not only causes serious psychological and physical pain to patients and nurses, but also brings enormous social and economic pressure to individuals and considerable losses to the global health economy [3].

Adiponectin (ADIPO) is an adipocytokine secreted mainly by adipocytes, first described in 1995 [4], [5]. It is found to be negatively correlated with visceral adiposity [6]. The human ADIPO gene (*ADIPOQ*) was cloned by sequencing human adipose tissue cDNA library [7]. Human ADIPO consists of 244 amino acids with a relative molecular weight of 30 KD and is located on chromosome 3q27 [8,9]. The human chromosome 3q27 has been shown to be a region carrying a susceptibility gene for T2DM [10]. There are three types of ADIPO receptors (*ADIPOR): ADIPOR1* (abundantly expressed in skeletal muscle), *ADIPOR2* (expressed in liver tissue), and *T-cadherin* (predominantly found in the heart and arteries) [11].

Civitarese [12] et al. have revealed that *ADIPOR1* and *ADIPOR2* isoforms may be important therapeutic targets for improving insulin sensitivity in patients with T2DM or in individuals at risk of developing the disease. ADIPO has a variety of important biological functions, which may improve insulin sensitivity in insulin target tissues, modulate inflammatory responses, and plays a crucial role in oxidative stress, atherosclerotic processes, and the regulation of energy metabolism [13,14].

The molecular signal transduction of ADIPO is activated by AMP-activated protein kinase (AMPK), PPARα, and p38 mitogen-activated protein kinase (MAPK) signalling pathways [15]. Yoon [16] et al. have provided evidence that ADIPO enhances fatty acid oxidation in muscle cells by stimulating PPAR transcriptional activity via the sequential activation of AMPK and p38MAPK. AMPK is a serine/threonine protein kinase, known as the ‘energy receptor’, which plays a key role in the balance of energy metabolism in body [17,18]. PPARα governs the expression of numerous genes involved in nearly every single aspect of lipid metabolism, including fatty acid uptake, mitochondrial and peroxisomal fatty acid oxidation, ketogenesis, and formation and breakdown of triglycerides and lipid droplets [19]. P38MAPK is a type of mitogen activated protein kinases (MAPKs), it consists of 360 amino acids with a molecular weight of 38 KD [20]. The p38MAPK signalling pathway is the junction or common pathway of cellular signalling [21]. There are still many unknown problems in the signal transduction pathway of ADIPO, such as the upstream signal molecules of p38MAPK and AMPK are not clear. Existing studies have shown that adiponectin signalling pathway plays a regulatory role in insulin signalling pathway and can cause insulin resistance [10,22].

In this study, we explored the relationship between ADIPO signalling pathway and T2DM, to provide clues for further study of the pathogenesis of T2DM and to determine the possible drug targets.

## 2. Materials and methods

### 2.1. Study population

1092 T2DM cases and 1092 health controls were recruited according to the inclusion criteria. The patients came from 8 people’s hospitals including Maoming City, Shaoguan City, Dongguan Houjie, Shenzhen Longhua, Shenzhen Nanshan, Shenzhen Guanlan, Shenzhen Xixiang and Shenzhen Futian, as well as 10 endocrinology departments of Affiliated Hospital of Guangdong Medical College and Dongguan Shilong Boai Hospital. The case group was adopted the 1999 WHO diabetes diagnostic criteria. The control group was consisted of healthy people with non-type 2 diabetes diagnosed by the same diagnostic criteria at the same hospital at the same time as the case group. We matched the case group to the control group by region and age. Selection criteria for control group: (1) Age between 20 and 70, (2) No family genetic history of diabetes, (3) The medical history, physical examination, blood glucose examination and other biochemical results showed no abnormality.

### 2.2. Information collection and blood sample collection

The subjects were surveyed by qualified professional investigators, including general information such as age and gender. Height and weight are measured to calculate BMI. Blood pressure and heart rate are measured by an electronic sphygmomanometer. Endocrinology nurses collected 5 ml of peripheral blood from healthy subjects and patients respectively in the morning to detect clinical biochemical indicators including FPG, TC, TG, HDL-C, and LDL-C. In addition, 4 ml of peripheral blood of the subjects (2 ml per tube) was taken and anticoagulated with EDTA·k2 and stored at −80°C.

### 2.3. Data collation and database establishment

All completed questionnaires were uniformly coded, and all participants’ questionnaire information, physical examination, and clinical biochemical examination results were compiled. Use EpiData 3.1 software to build a database and enter data by double input. The entered data is checked by both manual and computer methods to ensure that the data has no logic errors and no entry errors.

### 2.4. DNA extraction

Subjects need to be fasted for 8 hours before blood collection by a professional nurse. Blood samples were treated with dipotassium dihydrogen ethylenediaminetetraacetate (EDTA-K2). Protease K was used for digestion, and DNA was extracted by salting out method.

### 2.5. Screening and typing of SNPs

A pathway map of the ADIPO signalling pathway was obtained from the KEGG database to identify 13 major genes. Their upstream and downstream 5kb regions using Hapoloview (ver.4.2). Then use FastSN to select 1–2 high scores tagSNP for each gene. Finally, 23 tagSNPs were selected from 13 genes. The SNPscan^TM^ multiple SNP typing technology was used to classify the selected labelled SNPs. The basic principle of this technique is to use the high specificity of ligase ligation reaction to realize the recognition of SNP locus alleles. Then by introducing non-specific sequences of different lengths at the end of the connection probe and by ligase addition reaction, the corresponding ligation products of different lengths were obtained. The ligation products were amplified by PCR with labelled fluorescent universal primers. The amplified products were separated by fluorescence capillary electrophoresis. Finally, the genotypes of each SNP site were obtained by electrophoresis analysis. In the Chinese population, the minimum MAF is 0.051 (rs2744537), the maximum is 0.442 (rs4982856), the relevant information of each SNP is shown in Table 1.

**Table 1.** Basic information of 23 tagSNPs selected from 13 genes in the ADIPO signalling pathway

Gene	Chr	Position_37	SNP	Region	Allele		MAF
					Minor	Major	
*ADIPOQ*	3	186,559,474	rs266729	5ʹupstream	C	G	0.300
	3	186,561,634	rs16861205	intron1	G	A	0.179
*ADIPOR1*	1	202,914,356	rs1342387	intron4	T	C	0.399
	1	202,922,040	rs12733285	intron1	C	T	0.069
*ADIPOR2*	12	1,889,823	rs767870	intron5	G	A	0.084
	12	1,896,956	rs1044471	3ʹUTR	C	T	0.427
*PPARA*	22	46,598,307	rs4823613	intron4	A	G	0.217
	22	46,621,994	rs5767743	intron7	T	C	0.237
*PCK1*	20	56,140,980	rs1042531	3ʹUTR	T	G	0.206
	20	56,131,216	rs11908628	5ʹupstream	A	G	0.266
*PCK2*	14	24,569,418	rs2301336	exon7	A	G	0.230
	14	24,563,212	rs4982856	5ʹupstream	T	C	0.442
*G6PC*	17	41,056,245	rs2593595	intron2	A	G	0.139
*ACC2*	12	109,643,645	rs2268388	intron18	G	A	0.144
*GLUT1*	1	43,399,686	rs3754219	intron2	A	C	0.401
	1	43,409,179	rs12718444	intron1	G	T	0.150
*GLUT4*	17	7,187,123	rs5435	exon4	T	C	0.352
	17	7,186,022	rs16956647	intron1	C	T	0.248
*CPT-1*	11	68,593,258	rs11228368	intron1	A	G	0.368
*RXRA*	9	137,259,992	rs11185660	intron1	T	C	0.144
	9	137,332,311	rs1045570	3ʹUTR	T	G	0.212
*RXRB*	6	33,162,215	rs2744537	5ʹupstream	A	C	0.051
	6	33,166,034	rs2076310	intron3	A	G	0.426

Abbreviation: Chr, chromosome number; SNP, single nucleotide polymorphism; MAF, minor allele frequency

### 2.6. Statistical analysis

In the process of comparing all variables between the case group and the control group, the normal quantitative data were expressed as (*x ± s*), and the counting data were expressed as the number of cases or percentage. The differences in continuous variables between the two groups were tested by Student’s *t-*test. Comparison of categorical variable data between the two groups was tested by *χ*^2^ test. Genotype and allele frequency were compared by *χ*^2^ test. Pearson chi-square test, Cochran-Armitage trend test, MAX3 and logistic regression were used to analyse the association between single SNP and T2DM; unconditional logistic regression was used to analyse haplotype in LD block; and SNPs set analysis based on logistic kernel machine regression was used to analyse pathway. All statistical analysis was performed by SPSS25.0, PLINK 1.07, R 2.14.2, Haploview 4.2, SNPstats and other statistical software packages.

## 3. Results

### 3.1 The baseline data

After excluding cases with missing information, 1,067 people in the case group and 1,054 people in the control group were included in the analysis. The average age, body mass index (BMI), FPG, TG, and LDL-C of the case group were higher than those of the control group, and the difference was statistically significant (*P* < 0.05). See Table 2 for details.

**Table 2.** Comparison of baseline data between case group and control group

Parameters	T2DM	Control	*t/χ*^2^	*P-*value
n	1067	1054	-	-
Gender (%)				0.080
Male	532(49.86)	532(50.47)	0.08	
Female	535(50.14)	522(49.53)		
Age (years)	59.71 ± 11.87	57.23 ± 10.41	5.12	<0.001
BMI (kg/m^2^)	24.60 ± 3.24	23.58 ± 3.33	7.15	<0.001
Heartrate (Times/minute)	76.40 ± 15.26	76.20 ± 10.92	0.35	0.682
Hypertension (%)	396(37.11)	380(36.05)	0.26	0.257
FPG (mmol/L)	10.46 ± 4.50	5.60 ± 1.60	33.22	<0.001
TC (mmol/L)	5.31 ± 1.59	5.43 ± 1.27	−1.92	0.056
TG (mmol/L)	2.24 ± 1.03	1.31 ± 0.96	21.51	<0.001
HDL-C (mmol/L)	1.35 ± 0.54	1.37 ± 0.42	−0.95	0.398
LDL-C (mmol/L)	2.73 ± 1.04	3.03 ± 0.65	−7.98	<0.001

Abbreviation: BMI, body mass index = body weight/(height*height); FPG, fasting plasma glucose; TC, total cholesterol; TG, triglyceride; HDL-C, high-density lipoprotein cholesterol; LDL-C, low density lipoprotein cholesterol.

### 3.2 SNPs typing results

The success rate of 23 SNPs was above 98%, and the minimum allele frequency was 0.016 and the maximum was 0.476. The Hardy-Weinberg equilibrium test shows that each point satisfies the Hardy-Weinberg equilibrium. The results showed that the SNPs loci in this study were representative of the population (*P* > 0.01). See Table 3 for details.

**Table 3.** 23 SNPs genotyping results for 13 genes in the ADIPO signalling pathway

Gene	SNP	Allele		Call Rate (%)	MAF	*P*_HWE_
		Minor	Major			
*ADIPOQ*	rs266729	G	C	98.40	0.247	0.561
	rs16861205	A	G	98.37	0.157	0.741
*ADIPOR1*	rs1342387	C	T	98.37	0.348	0.332
	rs12733285	T	C	98.37	0.078	0.833
*ADIPOR2*	rs767870	A	G	98.34	0.137	0.023
	rs1044471	T	C	98.37	0.393	0.122
*PPARA*	rs4823613	G	A	98.37	0.232	0.232
	rs5767743	C	T	98.37	0.218	0.999
*PCK1*	rs1042531	G	T	98.37	0.281	0.038
	rs11908628	G	A	98.34	0.282	0.261
*PCK2*	rs2301336	G	A	98.37	0.221	0.852
	rs4982856	C	T	98.37	0.476	0.952
*G6PC*	rs2593595	G	A	98.21	0.167	0.251
*ACC2*	rs2268388	A	G	98.29	0.241	0.058
*GLUT1*	rs3754219	C	A	98.37	0.366	0.643
	rs12718444	T	G	98.37	0.150	0.028
*GLUT4*	rs5435	C	T	98.37	0.350	0.059
	rs16956647	T	C	98.18	0.261	0.025
*CPT-1*	rs11228368	G	A	98.37	0.259	0.341
*RXRA*	rs11185660	C	T	98.37	0.168	0.122
	rs1045570	G	T	98.37	0.173	0.731
*RXRB*	rs2744537	C	A	98.32	0.016	0.998
	rs2076310	G	A	98.34	0.367	0.891

Abbreviation: SNP, single nucleotide polymorphism; MAF, minor allele frequency; *P*_HWE_, values of the Hardy–Weinberg test for each SNP.

### 3.3 Allele association analysis results

The results of allele association analysis are shown in Table 4. There was no significant difference in the sub-allele frequency of each SNP between the case group and the control group. After adding age, BMI, and other covariate corrections, the sub-allele frequency of each SNP in the case group and the control group still had no statistical difference.

**Table 4.** Results of association analysis between ADIPO signalling pathway allele and type 2 diabetes

Gene	SNP	Allele	non-diabetic controls			T2DM patients			*OR* (95% *CI*)	
			A	a	MAF	A	a	MAF	Observed	Adjusted
*ADIPOQ*	rs266729	C/G	1584	524	0.249	1582	552	0.259	1.06 (0.92–1.22)	1.07 (0.93–1.24)
	rs16861205	G/A	1763	345	0.164	1821	313	0.147	0.88 (0.74–1.04)	0.89 (0.75–1.05)
*ADIPOR1*	rs1342387	C/T	1398	710	0.337	1374	760	0.356	1.09 (0.96–1.24)	1.09 (0.96–1.24)
	rs12733285	C/T	1945	163	0.077	1970	164	0.077	0.99 (0.79–1.24)	1.02 (0.81–1.28)
*ADIPOR2*	rs767870	A/G	1826	282	0.134	1846	288	0.135	1.01 (0.85–1.20)	1.01 (0.84–1.20)
	rs1044471	C/T	1272	836	0.397	1278	856	0.401	1.02 (0.90–1.15)	1.01 (0.89–1.14)
*PPARA*	rs4823613	A/G	1610	498	0.236	1637	497	0.233	0.98 (0.85–1.13)	0.97 (0.84–1.13)
	rs5767743	T/C	1638	470	0.223	1665	469	0.220	0.98 (0.85–1.13)	0.99 (0.85–1.15)
*PCK1*	rs1042531	T/G	1522	586	0.278	1514	620	0.291	1.06 (0.93–1.21)	1.08 (0.94–1.23)
	rs11908628	A/G	1509	599	0.284	1510	622	0.292	1.04 (0.91–1.19)	1.05 (0.91–1.20)
*PCK2*	rs2301336	A/G	1665	443	0.210	1640	494	0.231	1.13 (0.98–1.31)	1.11 (0.96–1.29)
	rs4982856	C/T	1132	976	0.463	1107	1027	0.481	1.08 (0.95–1.22)	1.08 (0.95–1.22)
*G6PC*	rs2593595	A/G	1774	334	0.158	1757	375	0.176	1.14 (0.97–1.34)	1.18 (0.99–1.40)
*ACC2*	rs2268388	G/A	1610	492	0.234	1623	511	0.239	1.03 (0.90–1.18)	1.05 (0.91–1.21)
*GLUT1*	rs3754219	A/C	1323	785	0.372	1352	782	0.366	0.98 (0.86–1.10)	0.97 (0.86–1.10)
	rs12718444	G/T	1794	314	0.149	1820	314	0.147	0.99 (0.83–1.17)	0.97 (0.81–1.15)
*GLUT4*	rs5435	C/T	1367	741	0.352	1369	765	0.358	1.03 (0.91–1.18)	1.03 (0.90–1.18)
	rs16956647	C/T	1552	552	0.262	1581	551	0.258	0.98 (0.85–1.13)	0.97 (0.84–1.12)
*CPT-1*	rs11228368	A/G	1556	552	0.262	1587	547	0.256	0.97 (0.84–1.12)	0.98 (0.85–1.13)
*RXRA*	rs11185660	T/C	1757	351	0.167	1775	359	0.168	1.01 (0.86–1.19)	1.05 (0.88–1.24)
	rs1045570	G/T	1768	340	0.161	1754	380	0.178	1.13 (0.96–1.32)	1.14 (0.96–1.34)
*RXRB*	rs2744537	C/A	2073	35	0.017	2102	32	0.015	0.90 (0.56–1.46)	0.93 (0.57–1.52)
	rs2076310	G/A	1337	771	0.366	1353	779	0.365	1.00 (0.88–1.13)	0.96 (0.85–1.10)

Note: Bold type indicates *P* < 0.05.

### 3.4 Genotype association analysis results

There was no statistical difference in the genotype distribution of each SNP between the case group and the control group, as shown in Table 5.

**Table 5.** Comparison of genotype frequencies between case group and control group in the ADIPO signalling pathway

Gene	SNP	Genotype	non-diabetic controls	T2DM patients	*χ*^2^	*P-*value
*ADIPOQ*	rs266729	CC/CG/GG	591/402/61	580/422/65	0.636	0.728
	rs16861205	GG/GA/AA	735/293/26	774/273/20	2.418	0.299
*ADIPOR1*	rs1342387	CC/CT/TT	456/486/112	434/506/127	1.809	0.405
	rs12733285	CC/CT/TT	896/153/5	912/146/9	1.369	0.504
*ADIPOR2*	rs767870	AA/AG/GG	800/226/28	796/254/17	4.253	0.119
	rs1044471	CC/CT/TT	396/480/178	371/536/160	4.781	0.092
*PPARA*	rs4823613	AA/AG/GG	622/366/66	622/393/52	1.535	0.464
	rs5767743	TT/TC/CC	636/366/52	651/363/53	0.117	0.943
*PCK1*	rs1042531	TT/TG/GG	563/396/95	533/448/86	4.393	0.111
	rs11908628	AA/AG/GG	532/445/77	525/460/81	0.328	0.849
*PCK2*	rs2301336	AA/AG/GG	656/353/45	626/388/53	2.929	0.231
	rs4982856	CC/CT/TT	303/526/225	269/569/229	3.665	0.160
*G6PC*	rs2593595	AA/AG/GG	741/292/21	722/313/31	2.831	0.243
*ACC2*	rs2268388	GG/GA/AA	628/354/69	619/385/63	1.517	0.468
*GLUT1*	rs3754219	AA/AC/CC	419/485/150	438/476/153	0.456	0.796
	rs12718444	GG/GT/TT	773/248/33	772/276/19	5.187	0.075
*GLUT4*	rs5435	CC/CT/TT	429/509/116	428/513/126	0.350	0.839
	rs16956647	CC/CT/TT	558/436/58	583/415/68	1.767	0.413
*CPT-1*	rs11228368	AA/AG/GG	568/420/66	582/423/62	0.226	0.893
*RXRA*	rs11185660	TT/TC/CC	725/307/22	736/303/28	0.749	0.687
	rs1045570	GG/GT/TT	743/282/29	719/316/32	2.395	0.302
*RXRB*	rs2744537	CC/CA/AA	1019/35/0	1036/30/1	0.457	0.499
	rs2076310	GG/GA/AA	425/487/142	422/509/135	0.606	0.739

To further confirm whether each SNP is associated with T2DM, whether the probability of disease increases with the increase of the number of risk alleles in the genotype, we have made Cochran-Armitage trend test under different genetic models (additive model, codominant model, dominant model, recessive model and overdominant model). Rs1044471 was statistically significant in the overdominant model, *P*_obs_ = 0.030, and the *OR* of genotype CT relative to TT-CC was 1.21, 95% *CI* (1.02–1.43). Rs1042531 was statistically significant in the overdominant model, *P*_obs_ = 0.038, and the *OR* of genotype GT relative to TT-GG was 1.20, 95% *CI* (1.02–1.44). In the recessive model of rs12718444, TT genotype was a protective factor compared with GG-GT genotype, *P*_obs_ = 0.043, *OR *= 0.56, 95% *CI* (0.32–0.99). The results were shown in Table 6.

**Table 6.** Results of association analysis under five genetic models in the ADIPO signalling pathway

SNP	Additive	Codominant *OR* (95% *CI*)		Dominant	Recessive	Overdominant
	*OR* (95% *CI*)	1^a^	2^b^	*OR* (95% *CI*)	*OR* (95% *CI*)	*OR* (95% *CI*)
rs266729	1.06 (0.92–1.22)	1.07 (0.89–1.28)	1.09 (0.75–1.57)	1.07 (0.90–1.27)	1.06 (0.74–1.51)	1.06 (0.89–1.26)
rs16861205	0.88 (0.74–1.04)	0.88 (0.73–1.07)	0.73 (0.40–1.32)	0.87 (0.72–1.05)	0.76 (0.42–1.36)	0.89 (0.74–1.08)
rs1342387	1.09 (0.96–1.24)	1.09 (0.91–1.31)	1.19 (0.90–1.59)	1.11 (0.94–1.32)	1.14 (0.87–1.49)	1.05 (0.89–1.25)
rs12733285	0.99 (0.79–1.24)	0.94 (0.73–1.20)	1.77 (0.59–5.30)	0.96 (0.76–1.23)	1.78 (0.60–5.34)	0.93 (0.73–1.19)
rs767870	1.01 (0.85–1.20)	1.13 (0.92–1.39)	0.61 (0.33–1.12)	1.07 (0.88–1.31)	0.59 (0.32–1.09)	1.14 (0.93–1.40)
rs1044471	1.02 (0.90–1.15)	1.19 (0.99–1.44)	0.96 (0.74–1.24)	1.13 (0.95–1.35)	0.87 (0.69–1.10)	**1.21 (1.02–1.43)**
rs4823613	0.98 (0.85–1.13)	1.07 (0.90–1.29)	0.79 (0.54–1.15)	1.03 (0.87–1.22)	0.77 (0.53–1.11)	1.10 (0.92–1.31)
rs5767743	0.98 (0.85–1.14)	0.97 (0.81–1.16)	1.00 (0.67–1.48)	0.97 (0.82–1.16)	1.01 (0.68–1.49)	0.97 (0.81–1.16)
rs1042531	1.06 (0.93–1.21)	1.19 (1.00–1.43)	0.96 (0.70–1.31)	1.15 (0.97–1.36)	0.88 (0.65–1.20)	**1.20 (1.01–1.43)**
rs11908628	1.04 (0.91–1.19)	1.05 (0.88–1.25)	1.07 (0.76–1.49)	1.05 (0.89–1.25)	1.04 (0.75–1.44)	1.04 (0.87–1.23)
rs2301336	1.13 (0.98–1.31)	1.15 (0.96–1.38)	1.23 (0.82–1.86)	1.16 (0.98–1.38)	1.17 (0.78–1.76)	1.13 (0.95–1.36)
rs4982856	1.08 (0.95–1.22)	1.22 (1.00–1.49)	1.15 (0.90–1.47)	1.20 (0.99–1.45)	1.01 (0.82–1.24)	1.15 (0.97–1.36)
rs2593595	1.14 (0.97–1.34)	1.10 (0.91–1.33)	1.52 (0.86–2.66)	1.13 (0.94–1.36)	1.47 (0.84–2.58)	1.08 (0.90–1.31)
rs2268388	1.03 (0.90–1.18)	1.10 (0.92–1.32)	0.93 (0.65–1.33)	1.07 (0.90–1.28)	0.89 (0.63–1.27)	1.11 (0.93–1.33)
rs3754219	0.98 (0.86–1.10)	0.94 (0.78–1.13)	0.98 (0.75–1.27)	0.95 (0.80–1.13)	1.01 (0.79–1.29)	0.94 (0.80–1.12)
rs12718444	0.99 (0.83–1.17)	1.11 (0.91–1.36)	0.58 (0.32–1.02)	1.05 (0.87–1.27)	**0.56 (0.32–0.99)**	1.13 (0.93–1.38)
rs5435	1.03 (0.91–1.78)	1.01 (0.84–1.21)	1.09 (0.82–1.45)	1.02 (0.86–1.22)	1.08 (0.83–1.42)	0.99 (0.84–1.18)
rs16956647	0.98 (0.85–1.13)	0.91 (0.76–1.09)	1.12 (0.78–1.62)	0.94 (0.79–1.11)	1.17 (0.81–1.68)	0.90 (0.76–1.07)
rs11228368	0.97 (0.84–1.12)	0.98 (0.82–1.17)	0.92 (0.64–1.32)	0.97 (0.82–1.16)	0.92 (0.65–1.32)	0.99 (0.83–1.18)
rs11185660	1.01 (0.86–1.19)	0.97 (0.80–1.17)	1.25 (0.71–2.21)	0.99 (0.82–1.19)	1.26 (0.72–2.22)	0.97 (0.80–1.17)
rs1045570	1.13 (0.96–1.32)	1.16 (0.96–1.40)	1.14 (0.68–1.90)	1.16 (0.96–1.39)	1.09 (0.66–1.82)	1.15 (0.95–1.39)
rs2744537	0.90 (0.56–1.46)	0.84 (0.51–1.38)	–	0.87 (0.53–1.42)	–	0.84 (0.51–1.38)
rs2076310	1.00 (0.88–1.13)	1.05 (0.88–1.26)	0.96 (0.73–1.26)	1.03 (0.87–1.23)	0.93 (0.72–1.20)	1.06 (0.90–1.26)

Note: ^a^ Codominant 1, heterozygous mutant type vs. homozygous wild type; ^b^ Codominant 2, homozygous mutant type vs. homozygous wild type. Bold type indicates *P* < 0.05.

To control for confounding factors, covariates (Age, BMI, Sex, and FPG) were added to the different genetic models for adjusting (Table 7). Rs1044471 was not statistically significant under the five models. Rs1042531 was still statistically significant in the overdominant model, *P*_adj_ = 0.044, and the *OR* of genotype GT relative to TT-GG was 1.21, 95% *CI* (1.02–1.45), under the codominant model, TG genotype was a protective factor compared with TT genotype, *P*_adj_ = 0.044, *OR *= 1.21, 95% *CI* (1.01–1.45), and genotype GG was not statistically significant relative to genotype TT, *P*_adj_ = 0.101, *OR *= 0.98, 95% *CI* (0.71–1.36). Rs12718444 still had statistical significance in the recessive model, *P*_adj_ = 0.014, and TT genotype was still a protective factor compared to GG-GT genotype, *OR *= 0.49, 95% *CI* (0.27–0.88), and TT genotype was a protective factor compared to GG genotype in the codominant model, *P*_adj_ = 0.029, *OR *= 0.50, 95% *CI* (0.28–0.90).

**Table 7.** Adjusting covariate results under five genetic models in the ADIPO signalling pathway ^a.^

SNP	Additive	Codominant *OR* (95% *CI*)		Dominant	Recessive	Overdominant
	*OR* (95% *CI*)	1 ^b^	2 ^c^	*OR* (95% *CI*)	*OR* (95% *CI*)	*OR* (95% *CI*)
rs266729	1.08 (0.93–1.24)	1.06 (0.88–1.27)	1.18 (0.81–1.73)	1.07 (0.90–1.28)	1.16 (0.80–1.68)	1.04 (0.87–1.24)
rs16861205	0.88 (0.74–1.05)	0.90 (0.74–1.10)	0.74 (0.40–1.35)	0.89 (0.73–1.07)	0.76 (0.42–1.39)	0.91 (0.75–1.10)
rs1342387	1.09 (0.96–1.25)	1.08 (0.90–1.30)	1.20 (0.90–1.60)	1.10 (0.92–1.31)	1.15 (0.88–1.52)	1.04 (0.87–1.23)
rs12733285	1.01 (0.80–1.27)	0.98 (0.76–1.26)	1.62 (0.53–4.92)	1.00 (0.78–1.28)	1.62 (0.53–4.93)	0.97 (0.76–1.25)
rs767870	1.01 (0.84–1.20)	1.10 (0.89–1.35)	0.68 (0.37–1.28)	1.05 (0.86–1.29)	0.67 (0.36–1.25)	1.11 (0.90–1.36)
rs1044471	1.01 (0.89–1.15)	1.16(0.96–1.40)	0.95 (0.73–1.23)	1.10 (0.92–1.32)	0.87 (0.69–1.11)	1.18 (0.99–1.40)
rs4823613	0.97 (0.84–1.12)	1.06 (0.88–1.28)	0.78 (0.53–1.15)	1.02 (0.86–1.22)	0.76 (0.52–1.12)	1.09 (0.91–1.30)
rs5767743	0.97 (0.85–1.15)	0.97 (0.81–1.17)	1.03 (0.69–1.55)	0.98 (0.82–1.17)	1.04 (0.70–1.56)	0.97 (0.81–1.16)
rs1042531	1.07 (0.94–1.23)	**1.21 (1.01–1.45)**	0.98 (0.71–1.36)	1.17 (0.98–1.39)	0.90 (0.66–1.23)	**1.21 (1.02–1.45)**
rs11908628	1.04 (0.91–1.19)	1.07 (0.89–1.28)	1.06 (0.75–1.49)	1.07 (0.90–1.27)	1.03 (0.74–1.43)	1.06 (0.89–1.27)
rs2301336	1.13 (0.97–1.31)	1.14 (0.95–1.38)	1.15 (0.76–1.75)	1.14 (0.96–1.37)	1.10 (0.72–1.66)	1.13 (0.94–1.36)
rs4982856	1.09 (0.96–1.23)	1.23 (1.00–1.51)	1.14 (0.89–1.47)	1.20 (0.99–1.47)	1.00 (0.81–1.23)	1.16 (0.98–1.38)
rs2593595	1.17 (0.99–1.38)	1.14 (0.94–1.38)	1.68 (0.95–2.97)	1.17 (0.97–1.41)	1.61 (0.91–2.85)	1.12 (0.92–1.35)
rs2268388	1.05 (0.91–1.20)	1.10 (0.92–1.33)	0.99 (0.69–1.43)	1.09 (0.91–1.30)	0.96 (0.67–1.37)	1.10 (0.92–1.33)
rs3754219	0.98 (0.86–1.11)	0.93 (0.77–1.12)	0.98 (0.75–1.28)	0.94 (0.79–1.12)	1.02 (0.80–1.31)	0.93 (0.78–1.11)
rs12718444	0.97 (0.82–1.15)	1.12 (0.91–1.37)	**0.50 (0.28–0.90)**	1.04 (0.85–1.26)	**0.49 (0.27–0.88)**	1.14 (0.93–1.40)
rs5435	1.03 (0.91–1.18)	1.02 (0.85–1.22)	1.08 (0.81–1.44)	1.03 (0.86–1.23)	1.07 (0.81–1.40)	1.00 (0.84–1.19)
rs16956647	0.97 (0.84–1.12)	0.92 (0.76–1.10)	1.07 (0.73–1.56)	0.93 (0.79–1.11)	1.11 (0.77–1.61)	0.91 (0.76–1.09)
rs11228368	0.98 (0.85–1.13)	1.00 (0.84–1.20)	0.91 (0.63–1.32)	0.99 (0.83–1.18)	0.91 (0.63–1.31)	1.01 (0.85–1.21)
rs11185660	1.04 (0.88–1.22)	1.00 (0.82–1.21)	1.35 (0.76–2.41)	1.02 (0.85–1.24)	1.35 (0.77–2.40)	0.99 (0.82–1.20)
rs1045570	1.13 (0.96–1.33)	1.18 (0.97–1.43)	1.13 (0.67–1.90)	1.17 (0.97–1.41)	1.07 (0.64–1.81)	1.17 (0.97–1.42)
rs2744537	0.94 (0.57–1.52)	0.88 (0.53–1.45)	–	0.90 (0.55–1.49)	–	0.88 (0.53–1.45)
rs2076310	0.98 (0.86–1.12)	1.00 (0.83–1.21)	0.91 (0.69–1.20)	0.98 (0.82–1.17)	0.91 (0.70–1.17)	1.03 (0.86–1.22)

Note: ^a^ The adjusted covariates include Age, BMI, Sex, and FPG. ^b^ Codominant 1, heterozygous mutant type vs. homozygous wild type. ^c^ Codominant 2, homozygous mutant type vs. homozygous wild type. Bold type indicates *P* < 0.05.

We further applied the MAX3 robust test method to compare the results with those based on various genetic models. The robust test results are shown in Table 8. Each SNP was not statistically significant in the results given by the robust method.

**Table 8.** Results of the robust test of the MAX3 method

Gene	SNP	*χ*^2^	*P*-value
*ADIPOQ*	rs266729	0.794	0.694
	rs16861205	1.542	0.223
*ADIPOR1*	rs1342387	1.345	0.326
	rs12733285	1.050	0.586
*ADIPOR2*	rs767870	1.700	0.190
	rs1044471	1.342	0.332
*PPARA*	rs4823613	1.395	0.321
	rs5767743	0.316	0.943
*PCK1*	rs1042531	1.600	0.215
	rs11908628	0.564	0.843
*PCK2*	rs2301336	1.688	0.188
	rs4982856	1.835	0.144
*G6PC*	rs2593595	1.541	0.242
*ACC2*	rs2268388	0.813	0.638
*GLUT1*	rs3754219	0.608	0.794
	rs12718444	2.010	0.097
*GLUT4*	rs5435	0.582	0.816
	rs16956647	0.842	0.652
*CPT-1*	rs11228368	0.437	0.903
*RXRA*	rs11185660	0.815	0.700
	rs1045570	1.547	0.240
*RXRB*	rs2744537	0.994	0.997
	rs2076310	0.552	0.828

### 3.5 Linkage disequilibrium analysis and association analysis based on haplotype

**Figure 1.**
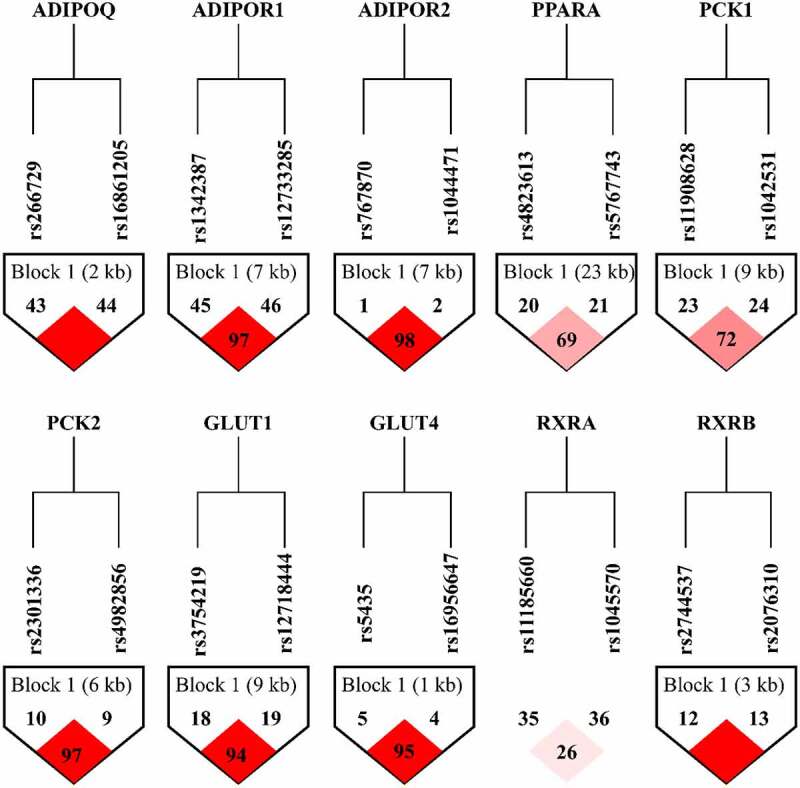
Results of LD analysis of 10 genes in ADIPO signalling pathway

Linkage disequilibrium (LD) analysis was performed between different sites within the same gene using Haploview 4.2 software. It was found that there was a linkage disequilibrium between the sites within 9 genes such as *ADIPOQ*, and the two loci in *RXRA* gene did not form blocks. Figure 1 shows the composition of the LD blocks of these 10 genes in turn.

LD analysis showed that the probability of co-existence of two alleles in the target gene was greater than the probability of co-occurrence due to random distribution in the population. Figure 1 shows the LD block composition of these 10 genes in turn, and two loci in *RXRA* gene did not form LD block.

Unconditional logistic regression analysis of haplotypes in LD blocks were performed using SNPstats online software. The analysis results were shown in Table 9. There were no statistically significant positive results for haplotypes in LD blocks in each gene.

**Table 9.** Results of haplotype unconditional logistic regression analysis of 9 genes LD block in ADIPO signalling pathway

Gene	SNP		SNP		Freq	*OR* (95% *CI*)	*P*-value
*ADIPOQ*	rs16861205	G	rs266729	C	0.591	1.00	–
		G		G	0.254	1.05 (0.91–1.22)	0.510
		A		C	0.155	0.90 (0.75–1.07)	0.241
*ADIPOR1*	rs12733285	C	rs1342387	C	0.652	1.00	–
		C		T	0.271	1.09 (0.95–1.26)	0.221
		T		T	0.076	1.08 (0.85–1.37)	0.542
*ADIPOR2*	rs1044471	C	rs767870	A	0.468	1.00	–
		T		A	0.398	1.01 (0.88–1.15)	0.933
		C		G	0.134	1.00 (0.83–1.21)	0.982
*PPARA*	rs4823613	A	rs5767743	T	0.714	1.00	–
		G		C	0.170	0.97 (0.83–1.15)	0.761
		G		T	0.065	0.99 (0.76–1.28)	0.910
		A		C	0.051	1.04 (0.78–1.38)	0.811
*PCK1*	rs1042531	T	rs11908628	A	0.450	1.00	–
		T		G	0.266	1.13 (0.96–1.32)	0.151
		G		A	0.262	1.15 (0.98–1.34)	0.087
		G		G	0.022	0.87 (0.51–1.51)	0.632
*PCK2*	rs2301336	A	rs4982856	C	0.525	1.00	–
		A		T	0.254	1.05 (0.90–1.22)	0.522
		G		T	0.218	1.12 (0.95–1.31)	0.171
*GLUT1*	rs12718444	G	rs3754219	A	0.486	1.00	–
		G		C	0.366	0.97 (0.85–1.11)	0.691
		T		A	0.145	0.97 (0.80–1.16)	0.713
*GLUT4*	rs16956647	C	rs5435	C	0.389	1.00	–
		C		T	0.351	1.03 (0.89–1.19)	0.733
		T		C	0.256	0.98 (0.84–1.15)	0.821
*RXRB*	rs2076310	G	rs2744537	C	0.634	1.00	–
		A		C	0.350	0.97 (0.85–1.10)	0.610
		A		A	0.016	0.92 (0.56–1.50)	0.733

### 3.6 SNPs – SNPs interaction results

**Figure 2.**
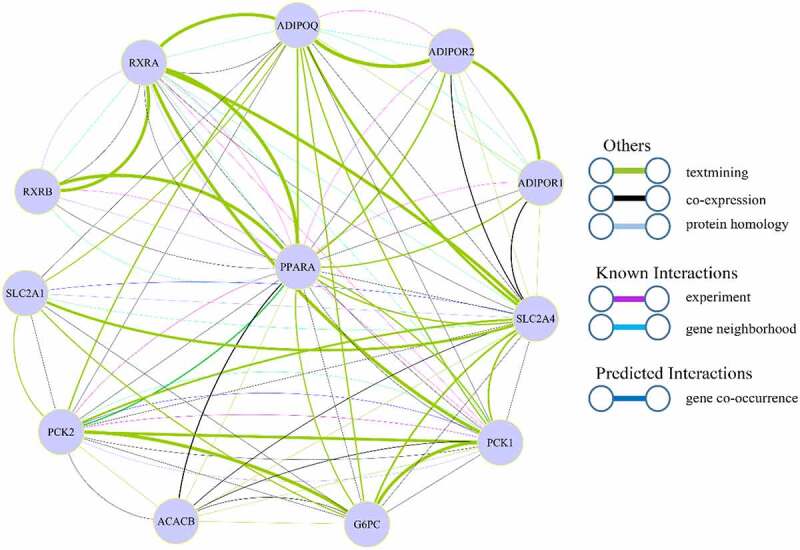
The interaction map of 13 genes in adipo signalling pathway

We uploaded 13 genes from the ADIPO signalling pathway to the STRING (Search Tool for the Retrieval of Interacting Genes/Proteins) tool. The interaction between proteins encoded by these genes was analysed and the results were shown in Figure 2.

(1) Network nodes represent proteins:Each node in the figure represents a protein due to variable splicing and post-transcriptional modification in eukaryotes. The letter marked on the node is the gene symbol of the corresponding gene.(2) Edges represent protein-protein associations: The line between nodes represents an interaction between two proteins. Associations are meant to be specific and meaningful, i.e. proteins jointly contribute to a shared function. Different colours correspond to different types of interactions. As you can see from the figure, there is more than one line between the two proteins, indicating that there are multiple interactions between the two proteins. Among all the correlations, there are both experimental verification and data prediction results. The width of the lines represents the degree of interaction between proteins. The bold lines represent greater interactions between proteins. The value (combined score) is larger.

### 3.7 Pathway analysis results

Four kernel functions, such as linear, linear-weighted, identical-by-state (IBS), and IBS-weighted, were used for SNPs set analysis based on ADIPO signalling pathway showing that there was no statistical significance whether covariates were added or not, *P* > 0.05, the results were shown in Table 10. The empirical *P* value obtained by the bootstrap method had no statistical significance.

**Table 10.** SNPs set analysis results based on ADIPO signalling pathway

Model	kernel	Q	*P-*value	resampling *P*
without covariates	linear	1757.38	0.157	0.141
	linear. weighted	25.61	0.252	0.234
	IBS	57.06	0.183	0.171
	IBS. weighted	28.95	0.257	0.239
with covariates	linear	1531.26	0.161	0.152
	linear. weighted	17.33	0.354	0.315
	IBS	49.84	0.161	0.148
	IBS. weighted	16.36	0.368	0.342

Abbreviation: IBS, identical-by-state

## 4. Discussion

In recent years, T2DM susceptibility and gene polymorphisms have been widely studied. Multiple gene SNPs in the adipocytokine signalling pathway have been shown to be significantly associated with the risk of developing T2DM, for example, 3 SNPs (rs10789038, rs2796498, and rs2746342) of the *PRKAA2* gene [23,24], 3 SNPs (rs1800206, rs4253776 and rs4253778) of the *PPARA* gene in the ADIPO signalling pathway [25] and 5 SNPs (rs1501299, rs17300539, rs2241766, rs266729 and rs16861194) of the *ADIPOQ* [26–29]. More and more evidences show that the study of gene polymorphisms is beneficial to the clinical diagnosis and treatment of diseases. Anna Maria Jung found that two SNPs (*SOS1* rs2888586 and *CDK4* rs2069502) were significantly associated with response to recombinant human growth hormone (rhGH) treatment [30]. Genetic variations are potentially suitable as predictive markers of rhGH treatment response in growth hormone deficiency. There is a study that has found an association between SNPs of some risk genes and the effect to antipsychotic therapy [31]. In the future, this means that patients may be able to select the most appropriate antipsychotic drug after testing these SNPs. At the same time, gene polymorphisms may provide clues for further study of the pathogenesis of T2DM and search for new drug targets.

Rs1042531 is located in the 3ʹUTR of *PCK1* gene on chromosome 20. *PCK1*, also known as cytoplasmic phosphoenolpyruvate pyruvate (PEPCK-C), is a multifunctional gene related to glycogen isogenesis, glycerol isogenesis, reproduction and female fertility, obesity and diabetes [32]. *PCK1* gene is highly expressed in adipocytes, and a radioactive imprint study indicates that *PCK1* in white adipose tissue is involved in glycerol xenobiotics [33,34]. Due to the lack of glycerol kinase in adipocytes, glycerol released by triglyceride degradation cannot be phosphorylated, and 3-phosphoglycerol necessary for free fatty acid re-esterification is a precursor substance derived from gluconeogenesis. Devine [35] et al. believe that *PCK1* is also the rate-limiting enzyme in glycerol xenobiotics. Overexpression of *PCK1* gene in adipocytes may be associated with obesity and insulin resistance. *PCK1* gene may be one of the important susceptibility genes related to T2DM. Any abnormality in the kinase product produced at the transcriptional or translational level may lead to diabetes. Vimaleswaran [36] et al. have found that *PCK1* gene polymorphism is not associated with obesity in European adolescents. Rees [37]et al. have discovered that rs1042531 is not associated with T2DM in South Asian populations. However, Jablonski [38] et al. have found that rs1042531 is associated with T2DM through GWAS research. This suggests that the locus is highly heterogeneous and varies by race or even by country. In this study, the association between *PCK1* rs1042531 and T2DM was further studied in Chinese Han population samples. Since the microRNA binds to the 3ʹUTR of the gene, the expression of the gene is regulated, and the rs1042531 site is located at the 3ʹUTR of the *PCK1* gene. We performed target microRNA prediction on the position of the rs1042531 site of the *PCK1* gene by the online software of miRNASNP (http://www.bioguo.org/miRNASNP/). We found that when the rs1042531 site T is mutated to a G base, the A base of the miR-1178 seed sequence region cannot be matched, thereby affecting the binding of miR-1178 to the *PCK1* gene and regulating the expression of the *PCK1* gene. Therefore, in the next functional experimental study, we will verify by experimental methods such as the construction of luciferase reporter vector.

Rs12718444 is located in the first intron region of *GLUT1* gene on chromosome 1. *GLUT1* is an important member of the GLUTs family, providing many cells with their basic glucose requirements, and it is a major transporter across the blood–brain barrier [39]. Because T2DM is characterized by persistent and abnormal extracellular hyperglycaemia [40], the relationship between them may be very close. Up to now, there is no report on rs12718444, so it needs to be validated by an independent population. Because the rs12718444 locus is located in the intron region of the gene, its function is unknown, it may be linked with other nearby gene SNPs or may affect the splicing of mRNA, thus affecting the function of proteins, which need to be further verified in subsequent studies. ADIPO has a protective effect on liver dysfunction in obesity,T2DM,and other insulin resistance states, and *ADIPOR2* is mainly expressed in liver [41]. The common SNPs in *ADIPOR2* (rs1044471) were associated with differences in liver function in the population. The human body may be able to increase circulating ADIPO through some negative regulation, thereby ameliorating the *ADIPOR2* gene variant (rs1044471) resulting in a decrease in insulin sensitivity [42].Our findings also proved that *ADIPOR2* rs1044471 may be related to the occurrence and development of T2DM, which further supported the research results of Martine Vaxillaire [43].

The premise of this study is that existing studies have found that ADIPO is closely related to energy metabolism and susceptibility to type 2 diabetes, while the specific function of ADIPO signal transduction pathway in T2DM is still unclear. According to our research results, it is found that some single nucleotide polymorphisms (ADIPOR2 rs1044471, PCK1 rs1042531, GLUT1 rs12718444) in the adiponectin signalling pathway may be associated with T2DM.Linkage disequilibrium analysis and haplotype-based association analysis showed that there was a linkage disequilibrium between the two loci in 9 genes such as *ADIPOQ* in the pathway. This is a preliminary independent sample verification for Chinese Han population, and its results can provide clues to whether ADIPO has a difference in correlation with T2DM due to ethnic heterogeneity. Therefore, it provides a partial research basis for further studying the pathogenesis of T2DM and looking for possible drug targets. We will also analyse the molecular mechanisms in subsequent studies to clarify the pathogenesis of diabetes from a genetic point of view.

In this study, 1067 subjects were included in the case group and 1054 subjects in the control group. The sample size is medium. In consideration of bias, cases from ten different hospitals were selected, and the samples were representative. However, the heterogeneity of different races was considered because the sample of this study is the only the Han population in Guangdong Province. The following cases of different races can be selected and the sample size can be increased to improve the credibility of the conclusion.

## 5. Conclusions

According to our research results, it is found that some single nucleotide polymorphisms (ADIPOR2 rs1044471, PCK1 rs1042531, GLUT1 rs12718444) in the adiponectin signalling pathway may be associated with T2DM.

## Data Availability

The data that support the findings of this study are openly available in ‘figshare’ at https://doi.org/10.6084/m9.figshare.15104142. The more detail of this study is available from the corresponding author upon reasonable request.
